# Special Issue “Hormone Signaling in Human Health and Diseases”

**DOI:** 10.3390/ijms27114956

**Published:** 2026-05-29

**Authors:** Antonella Muscella

**Affiliations:** Department of Biological and Environmental Science and Technologies (DiSTeBA), University of Salento, 73100 Lecce, Italy; antonella.muscella@unisalento.it

Complex interactions between hormones, which exert specific biological activities in their target tissues, are responsible for energy production and metabolism, somatic growth and development, reproduction, and the body’s ability to respond to internal and external stimuli [1]. Dissociation or dysfunction of hormonal signaling is a pathogenic mechanism underlying numerous clinical conditions, including metabolic disorders, reproductive dysfunction, and cardiovascular disease [2,3].

Genetic variations in hormone synthesis, receptor sensitivity, and regulatory genes create a complex and multifaceted regulatory landscape that governs endocrine resilience and vulnerability [4]. Kallmann syndrome is a congenital developmental disorder characterized by hypogonadotropic hypogonadism and anosmia, resulting from incomplete embryonic migration of gonadotropin-releasing hormone (GnRH)-producing neurons and olfactory neurons [5]. The cause of Kallmann syndrome is genetic and can result from numerous different mutations, the most common being in the KAL1 (ANOS1) and FGFR1 genes. Still, approximately 35–45% of cases are not explained by the currently identified genetic abnormalities [6,7]. Soejima et al. [8] reported an adult patient with Kallmann syndrome harboring a novel FGFR1 variant (c.2197_2199dup, p. Met733dup). In vitro studies in human granulosa cells demonstrate that this variant impairs FGFR1 signaling by downregulating SPRY2 and alters the expression of key steroidogenic enzymes, including StAR and P450arom, indicating a direct effect on gonadal steroidogenesis in addition to central hypogonadism. This finding strengthens the understanding that some FGFR1 variants may contribute to latent primary hypogonadism in KS [8].

Lifestyle habits (including diet, physical activity, sleep, and stress management) significantly influence hormonal function and balance, acting as crucial regulators of the endocrine axis [9]. Physical activity, a powerful and complex regulator, has effects that can promote or compromise hormonal health depending on the intensity, duration, and frequency of exercise.

Ghrelin, a stomach-derived peptide that primarily regulates food intake, appetite, and energy homeostasis, also functions to regulate growth hormone (GH) secretion; in turn, GH influences energy balance and fat metabolism [10]. Ghrelin and GH are involved in metabolic adaptations to exercise, with GH known for its role in stimulating muscle growth and fat oxidation [11].

Since circulating ghrelin levels are modulated by body weight, and physical training is one of the most useful tools for body weight control. Abassi et al. [12] evaluated the effects of acute and chronic exercise on circulating ghrelin and growth hormone (GH) levels. Short-term acute exercise (<60 min) generally does not alter total ghrelin concentrations, independent of intensity or GH secretion. In contrast, long-term chronic aerobic or combined training (≥12 weeks) increased ghrelin levels, particularly in overweight or obese individuals, likely through improvements in body composition, metabolic adaptations, and reductions in oxidative stress. GH responses were more consistently intensity-dependent, with higher-intensity exercise eliciting marked post-exercise increases [12].

These findings support recent research highlighting that hormones (such as GH, insulin, leptin, thyroid, ghrelin and sex hormones) act synergistically, receiving metabolic signals from various tissues and coordinating responses in terms of metabolism, growth, and fat distribution, in both physiological and pathological conditions [13,14].

In chronic kidney disease (CKD), this integrative role is exemplified by leptin, a hormone primarily produced by adipose tissue, which, through neurohormonal mechanisms, serves as a crucial link between energy stores, hormonal status, inflammation, and vascular function [15]. Rusu et al. [16] demonstrate that, in CKD patients, leptin levels are closely associated with adipose tissue mass, testosterone concentrations, and markers of vascular dysfunction, such as nitroglycerin-mediated vasodilation and brachial–ankle pulse wave velocity [16]. In both pre-dialysis and hemodialysis (HD) patients, increased adipose tissue mass leads to hyperleptinemia, which is inversely correlated with testosterone levels and directly with inflammatory markers, including high-sensitivity C-reactive protein (hs-CRP) and white blood cell count [16]. These findings underscore the multifaceted role of leptin: in addition to its classic effects on appetite regulation [17], leptin integrates signals from fat mass, inflammatory status, and sex hormones, thus influencing muscle composition, vascular smooth muscle function, and potentially cardiovascular risk [18,19].

Similarly, testosterone emerges as a potent modulator of fat-free mass and protein metabolism [20].

In CKD patients, testosterone deficiency is a prevalent condition, affecting 40–60% of hemodialysis patients, and is associated with significant metabolic and physical decline [21]. Furthermore, in CKD patients, testosterone levels correlate positively with hemoglobin, lean body mass, and markers of protein metabolism, highlighting its anabolic role [16]. Interestingly, the study shows sex-specific effects: while testosterone in male hemodialysis patients is primarily related to hemoglobin and vascular smooth muscle dysfunction, in the pre-dialysis phases, the correlations with metabolic and nutritional markers are less pronounced [16]. The correlations between metabolic hormones and nutritional or metabolic markers are less pronounced in pre-dialysis women than in men, but the systemic impact of leptin and inflammation remains significant. In women undergoing hemodialysis, leptin remains strongly correlated with adipose mass and inflammatory markers (hs-CRP, white blood cells), as in men. Endogenous testosterone, naturally lower than in men, shows weaker correlations with hemoglobin and vascular smooth muscle dysfunction, but may proportionally influence lean mass and body composition.

Thus, while in men it is mainly testosterone that modulates hemoglobin and vascular function, in women, leptin, through its effect on inflammation, represents the main coordinator of metabolic and tissue changes, thus playing a greater role in determining vascular dysfunction and cardiovascular risk [16].

The interaction between hormones and body composition is not limited to chronic kidney disease. In a study by Morelli et al. [22], in estrogen receptor-positive (ER+) breast cancer cells, androgens modulate the expression and nuclear and mitochondrial localization of the pro-apoptotic protein BAD (Bcl-2 cell death agonist) [22]. This regulation of BAD illustrates how hormones can coordinate protein expression [23], and even intracellular signaling and organelle function in response to environmental and metabolic stimuli [22]. Thus, in ER+ breast cancer cells, androgen signaling regulates mitochondrial and apoptotic pathways, highlighting context-specific hormonal control of cell fate [22].

Moreover, sex-specific differences in hormone signaling are evident in both studies. In CKD, women undergoing HD with higher adipose tissue mass exhibit stronger leptin-mediated vascular dysfunction than men [16]. In breast cancer, AR signaling exerts protective effects in ER+ cells, which predominantly affect female physiology [22].

Together, these findings emphasize the importance of sex and tissue context in interpreting hormonal effects, highlighting the need for personalized approaches in hormone-based therapies [24].

Leptin in CKD, androgen signaling in breast cancer, and ghrelin in metabolic regulation illustrate a shared principle: hormones orchestrate cellular survival, proliferation, and function, producing systemic or tissue-specific effects depending on the physiological or pathological context [12,16,22]. These context-dependent actions are strongly influenced by receptor type, density, and subcellular localization [24,25].

**Figure 1:**
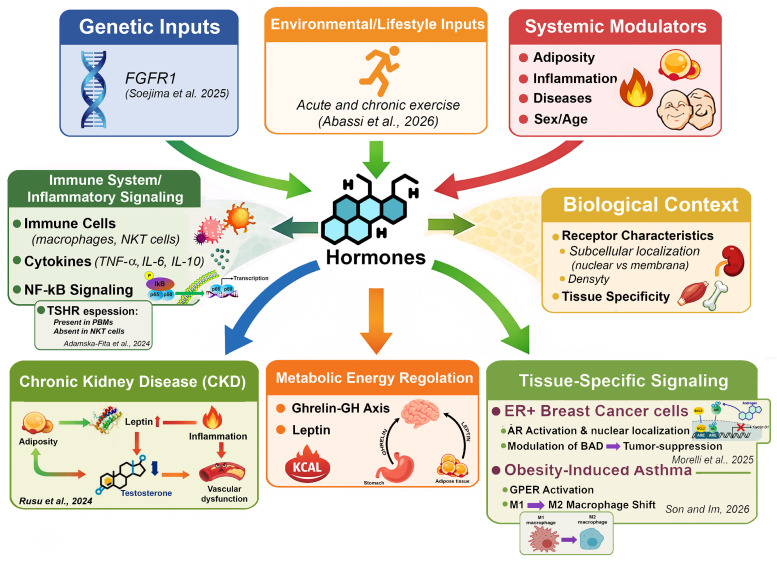
Hormones are integrators of intrinsic and extrinsic signals across physiological and pathological contexts. Lifestyle factors, adiposity, inflammation, and genetic background modulate hormonal signaling, whose effects depend on receptor type, density, and subcellular localization. Examples from chronic kidney disease, ER+ breast cancer, and metabolic regulation highlight how hormones coordinate metabolism, inflammation, tissue composition, and cell fate in a context-dependent manner [8,12,16,22,33,40].

These multidirectional interactions between genetic background, lifestyle factors, inflammation, and receptor-specific signaling are conceptually illustrated in Figure 1.

Intracellular AR is essential for BAD nuclear translocation and its anti-proliferative function, whereas membrane-bound AR, simulated with BSA-conjugated testosterone (T-BSA), can independently trigger apoptosis but does not influence BAD localization [22]. This illustrates how subcellular localization and receptor specificity determine t hormonal outcomes, a principle applicable across hormone systems [26].

In CKD, leptin exerts its effects through a combination of central and peripheral receptors [27]. In CKD, adipose tissue mass, hyperinsulinemia, and chronic inflammation amplify leptin signaling, leading to vascular and metabolic effects that are context- and sex-dependent [16]. Both examples illustrate that hormone action is not uniform across tissues, but rather is shaped by receptor expression, signaling pathway availability, and intracellular compartmentalization [26,27].

Another example of receptor- and context-specific hormonal action is provided by G1 protein-coupled estrogen receptor (GPER) signaling in a mouse model of obesity-induced asthma.

The altered adipokine profile in obesity, particularly elevated leptin, causes a shift toward M1 macrophage dominance, contributing to both systemic inflammation and airway hyperresponsiveness [28,29]. Chronic low-grade inflammation observed in obesity is a key factor exacerbating the pathological conditions of asthma [30]. Elevated levels of leptin stimulate the rise in IL-6 and TNF-α in adipocytes, directly associated with the production of IL-1β, IL-4, and IL-5 in the bronchial epithelium [31,32].

Activation of GPER with the selective agonist G-1 shifted lung macrophages from a pro-inflammatory M1 phenotype to an anti-inflammatory M2 phenotype, suppressed pro-inflammatory cytokines, and restored adipokine balance, effects that were reversed by a GPER antagonist [33]. The results suggest that activation of GPER could be a therapeutic option for obesity-induced asthma, targeting inflammatory pathways that promote M1 macrophage polarization.

The translational implications of these studies are significant. In CKD, leptin and testosterone levels may serve as biomarkers for nutritional status, vascular health, and disease progression, providing a pattern for patient stratification and targeted interventions. Pharmacologically reversing hyperleptinemia or testosterone deficiency can mitigate muscle atrophy, vascular dysfunction, and systemic inflammation [16]. In ER+ breast cancer, elevated AR and BAD expression correlates with improved overall survival (OS) and recurrence-free survival (RFS), suggesting that AR agonists or modulators of BAD localization could serve as adjunctive therapies [22]. In obesity-induced asthma, targeting GPER signaling can rebalance adipokine profiles and modulate inflammatory pathways, offering potential therapeutic benefits [33].

Overall, these examples demonstrate that understanding receptor- and context-specific hormonal mechanisms can inform personalized interventions aimed at optimizing tissue function, preventing disease progression, and improving clinical outcomes in diverse pathophysiological settings [3,34,35].

Hormones rarely act in isolation; their effects are influenced by and modulate other signaling pathways [3,34,35]. In CKD, leptin interacts with inflammatory mediators such as TNF-α, IL-6, and hs-CRP, with hyperleptinemia resulting in and amplifying inflammatory states via NF-κB activation, contributing to muscle catabolism and vascular dysfunction [14,36]. Similarly, ghrelin exerts anti-inflammatory effects by upregulating interleukin-10 and attenuating the release of pro-inflammatory cytokines in several tissues, illustrating its role as both a signaling molecule and a modulator of tissue homeostasis [37].

Together, these hormones exemplify complex, context-dependent feedback loops between the endocrine and inflammatory systems. These examples illustrate the broader principle that the endocrine and immune systems constantly interact, jointly regulating homeostasis, responses to infections, and autoimmunity [38]. Thyroid-stimulating hormone (TSH), for example, influences various immune cells through its receptor (TSHR), which is expressed on B lymphocytes, T lymphocytes, NK cells, monocytes, and dendritic cells [39].

Recent evidence indicates that TSHR is absent on natural killer T (NKT) cells, which are crucial for bridging innate and adaptive immunity and for modulating autoimmune responses [40]. Analysis of peripheral blood mononuclear cells from patients with benign nodular thyroid disease, with or without autoimmune thyroid disease (AITD), revealed TSHR expression in total PBMCs (2.77% TSHR+ cells) but not on NKT cells, regardless of disease status [40]. The absence of TSHR expression on NKT cells in patients with and without AITD suggests that contrary to previous hypotheses regarding the involvement of NKT cells in the pathophysiology of AITD [41], these cells do not directly participate in TSHR-mediated thyroid autoimmunity. However, given that the relationship between the number of NKT cells in peripheral blood and TSH levels has been documented, the involvement of NKT cells in the development and progression of AITD at the tissue level of the thyroid cannot be ruled out [42].

Collectively, the studies in this Special Issue underscore a central principle: hormones function as systemic integrators of intrinsic and extrinsic signals, coordinating metabolic, inflammatory, and proliferative pathways in a context-dependent manner. Their pleiotropic actions extend beyond classical roles, linking energy balance, immune regulation, tissue remodeling, and cellular homeostasis. However, much of the current evidence remains observational or preclinical, highlighting the need for mechanistic, tissue-specific, and sex- and age-informed research. A more integrated, multi-pathway therapeutic approach may therefore offer improved strategies for targeting hormone-mediated diseases across metabolic, reproductive, and cardiovascular contexts.
